# Immune Response to SARS-CoV-2 in an Asymptomatic Pediatric Allergic Cohort

**DOI:** 10.3390/antib10020022

**Published:** 2021-06-02

**Authors:** Nathan L. Marsteller, Diana J. Fregoso, Tricia L. Morphew, Inderpal S. Randhawa

**Affiliations:** 1 The Translational Pulmonary and Immunology Research Center (TPIRC), Long Beach, CA 90806, USA; dfregoso@tpirc.org (D.J.F.); docrandhawa@tpirc.org (I.S.R.); 2 MemorialCare Health System, Long Beach, CA 90806, USA; 3 Pediatric Pulmonology Division, Miller Children’s Hospital, Long Beach, CA 90806, USA; 4 Morphew Consulting, LLC, Bothell, WA 98021, USA; tricia@morphewconsulting.com; 5 Irvine School of Medicine, University of California, Irvine, CA 92617, USA

**Keywords:** antibody, atopy, infectious disease

## Abstract

Disease-specific COVID-19 pediatric comorbidity has not been studied effectively to date. Atopy and food anaphylaxis disease states require improved characterization of SARS-CoV-2 infection risk. To provide the first such characterization, we assessed serum samples of a highly atopic, food anaphylactic, asymptomatic pediatric cohort from across the US during the height of the pandemic. From our biobank, 172 pediatric patient serum samples were characterized specific to atopic, food anaphylactic, and immunologic markers in the US at the beginning of the pandemic, from 1 February to 20 April 2020. Clinical and demographic data were further analyzed in addition to sample analysis for SARS-CoV-2 IgM and IgG ELISA. SARS-CoV-2 antibody results were positive in six patients (4%). Nearly half of the pediatric patients had a history of asthma (49%). Total IgE, total IgG, and IgG1-3 were similar in those positive and negative to SARS-CoV-2. Median total IgG4 in the SARS-CoV-2 positive group was nearly three times (*p*-value = 0.02) that of the negative group. Atopy controller medications did not confer additional benefit. Our data suggest that food anaphylaxis and highly atopic children are not at increased risk for SARS-CoV-2 seropositivity. This specific population appears either at equal or potentially less risk than the general population. Total and specific IgG4 may be a novel predictor of SARS-CoV-2 infection risk specific to the allergic pediatric population.

## 1. Introduction

Coronavirus disease 2019 (COVID-19) is a respiratory disease caused by Severe Acute Respiratory Syndrome Coronavirus 2 (SARS-CoV-2). The disease was first reported in the Hubei province of China in December 2019. Since then, the virus has resulted in a pandemic declared on 11 March 2020 and has infected over 137 million people. Over 2.9 million have died worldwide, with the US holding the largest share of 31 million infections and 558,000 deaths as of 15 April 2021 (World Health Organization (WHO)). Once the virus infects a human, it can be transmitted from person to person without an intermediary [1,2]. The infection mechanisms of the novel SARS-CoV-2 and complexity of the pediatric immune system hinder our understanding of the COVID-19 disease state in children and adolescents. Identification of disease-specific pediatric comorbidity risk factors in COVID is limited due to the nature of research restrictions during the pandemic. Clinical presentation of SARS-CoV-2 infection spans from a lack of symptoms to fatal illness. Although all ages are susceptible to the virus, it more severely affects patients 65 years or older. Children have been reported to have various symptoms, but studies show as many as 45% of pediatric cases are asymptomatic [3]. A minority of the infected pediatric population has had an illness progress to multisystem inflammatory syndrome (MIS-C), resulting in severe complications possibly due to SARS-CoV-2 infection [4]. Of the global pediatric epidemiological studies specific to COVID-19, the lowest reported pediatric infection rate was 0.39% [5] in China at the beginning of the pandemic, while the highest reported was 34.1% [6]. At the time of publication, the reported infection rate in the pediatric population is 4.7% (American Academy of Pediatrics, *Children and COVID-19: State Data Report*, updated 8 April 2021).

Humoral response against SARS-CoV-2 is characterized by IgM, IgA, and IgG productivity as early as four days post symptom onset. IgM peaks around the second or third week and almost disappears by the seventh week while IgG persists [7]. When comparing the antigen used to detect IgM and IgG on ELISA, the spike protein has improved sensitivity compared to the nucleocapsid protein [8] and better specificity [7]. Antibody responses to the spike ELISA, spike-RBD ELISA, and ACE2 blockade of binding ELISA correlated with neutralizing IgG antibody titers [9]. Therefore, the presence of anti-spike IgG has the potential for neutralizing activity. There is evidence of preexisting T cell responses in people that have not been infected by SARS-CoV-2 [10]. It seems these responses can persist after infection [11]. If there is a T cell response from a circulating non-COVID-19 coronavirus that can lead to COVID-19 protection, that would be in line with what you may expect from circulating “common cold” coronaviruses, which can reinfect people but seem to result in milder symptoms [12]. This may help explain why children have milder symptoms when infected with SARS-CoV-2, as a child’s behavior and environment often lead to infection by various common colds [6].

The pediatric population thus far has been assessed for COVID infection in a largely post hoc approach for a snapshot of the early stages of the pandemic. Comorbidity in the pediatric population has not been studied effectively to date. In this study, we utilize serological testing to elucidate the antibody responses of a highly atopic, food anaphylactic pediatric cohort in the US from 1 February to 20 April 2020. Identification of this unique cohort of asymptomatic pediatric patients during the initial phase of the COVID-19 pandemic elucidates potential contributing factors of COVID infection risk. Analysis of clinical, molecular, immunologic, and geographic data markers serve to develop the first description of COVID-19 disease risk in an atopic cohort.

## 2. Methods

### 2.1. Study Design

Between 1 February and 20 April 2020, 250 whole blood samples were collected from children and adolescents prior to treatment at the Translational Pulmonary and Immunology Research Center (TPIRC) in California. All patients were enrolled as part of a food anaphylaxis treatment cohort. At the time of enrollment, all patients were evaluated by a physician and noted to be asymptomatic of any acute disease process. No patient at the time of enrollment had received any form of food immunotherapy. Patients were screened to meet the following criteria at time of blood draw: between the ages of 4 and 18 years, residence in the US, and asymptomatic. Out of 250, 172 patients met the requirements.

TPIRC houses an IRB-approved biobank of blood samples drawn from patients enrolled in the Tolerance Induction Program for the diagnosis and treatment of food anaphylaxis. Patients enrolled in the study lived in various locations in the United States. The study to obtain and analyze the blood samples specific to COVID-19 was reviewed and approved by the Advarra Institutional Review Board (IRB #PRO00043361). The blood samples were processed to isolate serum by centrifugation. The serum samples were aliquoted into cryotubes and stored at −80 °C prior to analysis.

### 2.2. ELISA

Enzyme Linked Immunosorbent Assay (ELISA) was used for the qualitative detection of anti-SARS-CoV-2 IgM and IgG (InBios International, Inc. COVE-G; COVE-M). Briefly, patient serum diluted 1:100 was added in duplicate wells to the SARS-CoV-2 spike protein-coated plates and incubated for 1 h at 37 °C. Plates were washed with phosphate buffered saline with Tween 20, and then IgM or IgG conjugate solution was added and incubated for 30 min at 37 °C. Plates were washed once more. TMB (3,3′,5,5′-Tetramethylbenzidine) substrate was added and plates were incubated for 20 min in the dark at room temperature (22.7 °C). The reaction was stopped with 1 N sulfuric acid. Optical density was read at 450 nm on the Azure Biosystems Ao Microplate Reader and evaluated according to the InBios Quality Control and Interpretations of Results.

Positive samples were determined by calculating the immunological status ratio (ISR) per the InBios instructions for use. The ISR was calculated by taking the average optical density (OD) of each sample and dividing it by the average OD of the plate’s cut-off control. An ISR value ≥1.1 is a positive value. ISR values between 0.9 and 1.1 are inconclusive and values ≤0.9 are negative.

### 2.3. Statistical Analysis

Demographic and clinical data were presented as percentage with defined trait, mean and SD, or median with interquartile range (IQR) when distribution showed departure from normality. Comparisons of means and medians between SARS-CoV-2 infection groups for continuous variables, e.g., immune response parameters and sIgE levels for food allergens and aeroallergens (sIgE levels were measured using ImmunoCap (Phadia, Kalamazoo, MI, USA) fluorescent enzyme assay in our lab), were performed using the independent *t*-test and Mann–Whitney U test, respectively. Factors defined on a categorical scale (e.g., +/− to specific allergen) were compared in terms of percentage positive to SARS-CoV-2 using Fisher’s exact test. All tests were 2-tailed with a level of significance set at *p* < 0.05. Statistical analyses were performed using SPSS version 18.0 0 (IBM Corp., Armonk, NY, USA) and R version 3.6.1. (R Foundation for Statistical Computing, Vienna, Austria).

## 3. Results

From 1 February to 20 April 2020 a total of 172 children and adolescents enrolled for clinical care at TPIRC with no active treatment prior to blood draw and no recent illness for the two previous months prior to collection. SARS-CoV-2 infection status was conclusive in 171 patients. Test results were positive in six patients (4%) who were asymptomatic at time of blood draw (Table 1). In the 171 patients, average age was 8.8 years (SD = 4.0) and the majority were less than 12 years of age (73%), male (57%), and Non-Hispanic (87%). Nearly half of the pediatric patients initiating care at TPIRC had a history of asthma (49%) with representation from all US regions (Northeast 11%, Midwest 9%, South 13%, and West 68%). Recent medication use in patients was predominately OCS (69%) and SABA (26%) with 16% reporting ICS use and 6% Sublingual Immunotherapy (SLIT) therapy.

Positive status to SARS-CoV-2 presented in higher proportion among adolescents (6%), Hispanic patients (9%), and those reporting SLIT therapy (10%) and LABA use (17%), although significance was not detectable (*p* > 0.05) (Table 1). Tracking with the spread of the virus during the initial months of the outbreak, none of the 15 patients from the Midwest were positive, and the highest percentage came from the Northeast (6%). Median IgG OD450 and IgG4 levels were significantly higher in patients positive to SARS-CoV-2, *p* < 0.05 (Table 2). Although average IgM OD450 was also higher, (0.21 vs. 0.09) in patients that were IgG-positive so too was the IgM OD450 variation (SD = 0.28) compared to those negative (SD = 0.04), *p* < 0.05, producing non-significant differences in average values between infection groups, *p* = 0.36. Distribution profiles for total IgE, total IgG, and IgG1-3 were similar in those positive and negative to SARS-CoV-2.

**Figure 1:**
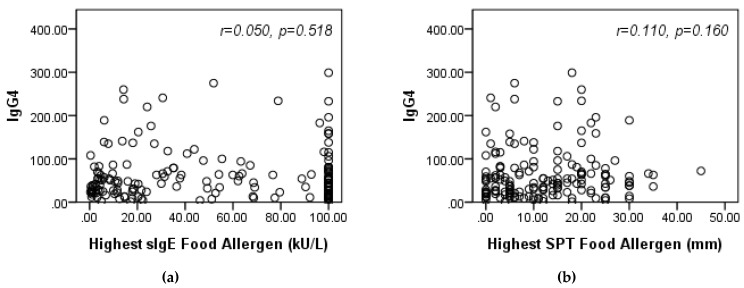
Scatterplots display highest sIgE (**a**) and skin prick test (SPT) (**b**) in patients with at least one positive food allergen (sIgE ≥ 0.35 kU/L) against immune response parameter IgG4 (*r* = Spearman’s correlation coefficients), *n* = 167.

Patient sensitivity to each of six common peanut and nut allergens did not significantly relate to SARS-CoV-2 positivity (*p* > 0.05); an exception was observed in almond allergic children where median sIgE was higher in those SARS-CoV-2-negative compared to positive (median=1.9 [IQR 0.9, 4.6] vs. 0.7 [0.6, 0.8]), *p* = 0.03, (Table 3). In the examination of nine common aeroallergens, a much higher percentage of patients who were allergic vs. non-allergic to mouse tested positive to SARS-CoV-2 (17% vs. 3%, *p* = 0.059), (Table 4). Conversely, no patients sensitive to cats and cockroaches were SARS-CoV-2 positive (*p* > 0.05). Although non-significant, the maximum sIgE across the nine aeroallergens was higher, on average, in the SARS-CoV-2-negative group compared to positive group (median [IQR]: 31.8 [13.7, 71.9] vs. 9.3 [7.9, 43.8], *p* = 0.20). This trend was similar to the one observed in children sensitive to almonds. In patients with at least one positive food allergen, immune response parameter IgG4 did not directly correlate to higher sensitivity (sIgE), *r*_s_ = 0.050, *p* = 0.52 or higher reactivity (skin prick test (SPT)), *r*_s_ = 0.110, *p* = 0.16 based on maximum values observed across food allergens, (Figure 1).

## 4. Discussion

Our study is the first description of demographic characteristics, medication use, and humoral response in an asymptomatic pediatric cohort seeking care for food anaphylactic disease in the US. We report 4% SARS-CoV-2 seroprevalence with a higher proportion coming from the Northeast, as would be expected, but also, prevalence in California patients indicates presence in California earlier than reported. There are inadequate studies on the characterization of antibody responses in pediatric patients. Current studies focus on severe COVID-19 disease in children and very few describe humoral response to SARS-CoV-2. This study is the first to demonstrate positive antibody results in asymptomatic patients during the early phase of the pandemic in an atopic pediatric cohort.

A primary goal of this study was to define comorbidity risk factors of COVID-19 in a food anaphylactic population. Atopy is a type 1 hypersensitivity disorder which is defined by a genetic tendency to develop allergies through an aberrant IgE-mediated response toward typically innocuous antigens. Due to the inclination toward T_H_2 cell-dependent immune responses in atopic individuals, they are generally at high risk for viral infection and severe illness [13,14]. In our characterized atopic cohort, risk of viral disease and infection is considered elevated. Many of our patients are on various types of atopic medication controllers and some received pollen SLIT. Despite these risk factors we are only seeing seropositivity for SARS-CoV-2 at 4%.

Perhaps the most implausible finding of SARS-CoV-2 infection is its reduced prevalence among chronic lung disease patients. In this pediatric cohort 49% had asthma. Asthma is a common comorbidity in food anaphylaxis patients. The rate of asthma in this demographic is an important consideration given SARS-CoV-2 proliferates in the lung. This is important as it demonstrates a higher risk population studied in the cohort. Atopic asthma and chronic obstructive pulmonary disease (COPD) do not seem to be significant risk factors for SARS-CoV-2 infection as the reported prevalence in SARS-CoV-2 infections is low when compared to other comorbidities [15]. Epithelial ACE2 expression in atopic individuals is low [16] while it is upregulated in individuals with COPD [17]. Though ACE2 expression is contradictory in these two disorders, they both appear to impart some protection from infection. In atopic individuals, this may be due to the low availability of ACE2, which SARS-CoV-2 uses for entry to the host cell. COPD patients have upregulated ACE2 expression but inhaled corticosteroids have been reported to downregulate ACE2 expression via suppression of type I interferon [18]. In addition, ACE2 expression was lower in SARS-CoV-2-negative children [19]. A subgroup of patients in our cohort were on inhaled corticosteroids at the time of analysis. There is some evidence that corticosteroids use in patients with COVID-19 is associated with reduced mortality compared to placebo or usual care [20]. It seems inhaled corticosteroid use downregulates ACE2 in COPD patients [18]. Indeed, active debate continues whether systemic corticosteroid use is appropriate in COVID-19 [21,22]. In our cohort, chronic use of inhaled corticosteroids did not offer additional prophylaxis in risk reduction in this population. Protection provided by low ACE2 expression is plausible and would help explain the low prevalence of SARS-CoV-2 infection among atopic patients.

Is there a variable or variables which accommodate the risk for acquisition of SARS-CoV-2? Baseline B cell antibody class switching to a preferential IgE class is clear in this cohort. IgE-mediated allergy specific to pollen and foods, total IgE, and other atopic factors did not uniquely correlate risk of SARS-CoV-2 seropositivity. Baseline IgG distribution is consistent with other atopic cohorts. Our subgroup analysis demonstrated atopy regardless of baseline antibody IgG class switch predilection was not correlated with SARS-CoV-2 seropositivity with one exception. The median total IgG4 in the SARS-CoV-2-positive group was nearly three times that of the negative group. IgG4-related disease (IgG4-RD) is a recently described systemic fibroinflammatory disease associated with elevated circulating levels of IgG4. No patients in our cohort displayed any symptoms of this condition [23]. The role of elevated IgG4 has been described in fine-tuning tolerance in IgE-mediated allergy [24]. IgG4 may play a role as a biomarker in the local inflammatory environment associated with lectin complement activation. Viral-associated endothelial injury is described as a key factor in lung disease associated with SARS-CoV-2. Narsoplimab, a high-affinity fully human immunoglobulin gamma 4 (IgG4), has been reported to block the lectin pathway by binding to mannan-binding lectin-associated serine protease-2 (MASP-2), which binds to COVID-19 N protein in disease progression. The results revealed by Rambaldi et al. provide a novel insight into COVID-19 immunological therapy via IgG4 complement inhibition [25]. Low serum IgG4 disease states have been well described. However, elevated serum IgG4 specific to viral infection risk is novel and requires further elucidation as a potential risk factor for SARS-CoV-2 infection.

Food anaphylaxis poses a life-threatening reaction upon antigen exposure. Food atopy, similar to other atopic diseases, appears to be less associated with SARS-CoV-2 infection and morbidity. If a food anaphylaxis child contracts SARS-CoV-2, the approach to treatment should fall under current guidelines based upon symptoms. However, food anaphylaxis patients are at rare risk of atopic vasculitis. Given COVID-19-associated vasculitis affiliation, a low clinical threshold to identify vasculitis is advised [26].

Our study has several limitations. The use of qPCR testing was not an option due to the use of previously stored serum samples. In our continued attempt to characterize pediatric COVID-19 in the allergic population, qPCR will be utilized for our prospective study. The clinical parameters of SARS-CoV-2 resulting in seropositive specific antibody response have yet to be clearly agreed upon. The population in our study was limited in size and geographic diversity. Atopy controller medications were limited in our cohort to antihistamines, intranasal corticosteroids, and less commonly inhaled corticosteroids. Recently, the use of a higher dose inhaled corticosteroid for active SARS-CoV-2 infection appeared to improve disease outcomes [27]. Hence, one drawback of our study is the lack of inhaled and intranasal corticosteroid controls in the study population. Finally, our study did not assess the mucosal physiology specific to atopy and SARS-CoV-2 epithelial ACE2 expression specific to our cohort. We will continue to study our atopic population with a future state enhanced focus on the in vivo mechanisms of the nasal respiratory mucosal interface.

## 5. Conclusions

Our study sought to assess if atopic children were at increased risk for infection by SARS-CoV-2. Overall, food anaphylaxis and highly atopic children do not appear at increased risk. This specific population appears at either equal or potentially less risk than the general population. In our study, 4% of our pediatric cohort in the US showed positivity for anti-SARS-CoV-2 IgM and/or IgG. It was not clear whether the use of controller medications in this population provided additional benefit, and this needs to be further studied. Our data suggest that total and specific IgG4 may be a novel predictor of SARS-CoV-2 infection risk specific to the allergic pediatric population.

## Figures and Tables

**Table 1:** Patient characteristics described in relation to positive SARS-CoV-2 status.

Overall		*n*	% Positive	*p*-Value ^a^
		171	4%	
Age ^b^				*p* = 0.35
4–11 years		124	2%	
12–18 years		47	6%	
Gender				*p* = 1.0
Female		73	3%	
Male		98	4%	
Ethnicity				*p* = 0.17
Hispanic		22	9%	
Non-Hispanic		149	3%	
History of Asthma				*p* = 0.44
No		87	2%	
Yes		84	5%	
Region:				*p* = 0.66
Northeast		18	6%	
Midwest		15	0%	
South		22	5%	
West		116	3%	
Medications:				
SLIT ^c^	Yes	10	10%	*p* = 0.31
	No	161	3%	
OCS ^d^	Yes	118	4%	*p* = 0.67
	No	53	2%	
SABA ^e^	Yes	45	2%	*p* = 1.0
	No	126	4%	
ICS+LABA	Yes	2	0%	*p* = 1.0
	No	169	4%	
ICS ^f^	Yes	28	4%	*p* = 0.98
	No	143	4%	
LABA ^g^	Yes	6	17%	*p* = 0.20
	No	165	3%	
LTRA ^h^	Yes	10	0%	*p* = 1.0
	No	6	4%	

^a^ Test of between group differences in distributions based on Fisher’s exact test. ^b^ Mean age did not significantly differ between + and − groups (9.0 (SD = 4.1) vs. 8.8 (SD = 4.0), respectively, *p* = 0.902). ^c^ Sublingual immunotherapy, ^d^ Oral corticosteroids, ^e^ Short-acting beta-agonists, ^f^ Inhaled corticosteroids, ^g^ Long-acting beta-agonists, ^h^ Leukotriene receptor antagonists.

**Table 2:** Immune response parameters described in relation to positive SARS-CoV-2 status.

	SARS-CoV-2		
Antibody Isotype	Positive *n* = 6 (3.5%)	Negative *n* = 165	*p*-Value ^a^
IgM OD450	0.21 (0.28)	0.09 (0.04)	*p* = 0.36 ^b^
IgG OD450	1.05 [0.81, 1.56]	0.08 [0.06, 0.12]	*p* < 0.001
Total IgE	334.5 [311.0, 387.0]	417.0 [188.0, 816.0]	*p* = 0.76
Total IgG	1075.2 (374.6)	1019.7 (267.5)	*p* = 0.62
IgG1	562.5 (229.3)	576.8 (169.7)	*p* = 0.84
IgG2	283.8 (137.9)	272.9 (115.7)	*p* = 0.82
IgG3	55.5 [46.0, 122.0]	58.0 [42.0, 76.0]	*p* = 0.69
IgG4	126.0 [64.0, 234.0]	44.0 [22.0, 75.0]	*p* = 0.02

^a^ Test of between group differences in distributions based on independent *t*-test when means reported, and Mann–Whitney U test when medians reported. ^b^ Equal variances not assumed as Levene’s test significant (*p* < 0.001). Results were reported in mean (SD) or median [IQR].

**Table 3:** Six common peanut and nut allergen parameters described in relation to positive SARS-CoV-2 status (*n* = 170 with allergen test results).

					In Patients + to Specific Allergen:		
Allergen	+	*n*	% SARS-CoV-2 Positive	*p*-Value ^a^	SARS-CoV-2 Positive sIgE, Median [IQR]	SARS-CoV-2 Negative sIgE Median [IQR]	*p*-Value ^b^
Peanut	Yes	142	4%	*p* = 1.0	38.3 [10.7, 100]	18.9 [3.0, 100]	*p* = 0.47
	No	28	4%				
Sesame	Yes	123	3%	*p* = 0.67	4.0 [1.1, 11.0]	1.6 [0.7, 11.2]	*p* = 0.45
	No	47	4%				
Cashew	Yes	98	5%	*p* = 0.24	18.1 [10.4, 23.3]	8.2 [2.5, 40.2]	*p* = 0.55
	No	72	1%				
Hazelnut	Yes	122	5%	*p* = 0.19	2.3 [1.1, 5.6]	3.5 [1.2, 8.2]	*p* = 0.61
	No	48	0%				
Pecan	Yes	74	3%	*p* = 0.70	6.7 [0.4, 13.0]	3.4 [1.1, 9.2]	*p* = 0.70
	No	96	4%		(only 2 patients)		
Almond	Yes	94	4%	*p* = 0.69	0.7 [0.6, 0.8]	1.9 [0.9, 4.6]	*p* = 0.02
	No	76	3%				
Allergic to any of above	Yes	158	4%	*p* = 1.0	28.2 [2.0, 100] ^c^	27.6 [6.7, 100] ^c^	*p* = 0.80
	No	12	0%				

^a^ Test of between group differences in distributions based on Fisher’s exact test. ^b^ Test of between group differences in distributions based on Mann–Whitney U test. ^c^ Distribution for highest level observed across positive allergens among the six types of nuts. + Food Allergen sIgE is positive if ≥0.35 kU.

**Table 4:** Aeroallergen sensitivity described in relation to positive SARS-CoV-2 status.

					In Patients + to Specific Aeroallergen:		
Allergen	+	*n*	% SARS-CoV-2 Positive	*p*-Value ^a^	SARS-CoV-2 Positive sIgE, Median [IQR]	SARS-CoV-2 Negative sIgE Median [IQR]	*p*-Value ^b^
Cat	Yes	56	0%	*p* = 0.18	----	20.2 [7.0, 45.8]	----
	No	114	5%				
Dog	Yes	72	6%	*p* = 0.40	9.0 [6.0, 26.9]	15.3 [6.9, 31.6]	*p* = 0.46
	No	98	2%				
Mouse	Yes	12	17%	*p* = 0.05	7.4 [5.5, 9.3]	8.0 [3.4, 29.8]	*p* = 0.83
	No	158	3%				
Cockroach	Yes	19	0%	*p* = 01.0	----	7.7 [4.1, 22.7]	----
	No	151	4%				
Dust Mites	Yes	47	2%	*p* = 1.0	Only 1 patient	30.3 [11.8, 99.8]	----
	No	123	4%				
Molds	Yes	57	2%	*p* = 0.66	Only 1 patient	14.9 [7.2, 24.9]	----
	No	113	4%				
Grass	Yes	53	3%	*p* = 1.0	40.1 [12.3, 67.9]	16.4 [7.5, 39.1]	*p* = 0.58
	No	107	4%				
Trees	Yes	73	3%	*p* = 0.70	13.9 [4.1, 23.6]	12.4 [6.2, 28.0]	*p* = 0.71
	No	97	4%				
Weeds	Yes	42	5%	*p* = 0.64	10.6 [5.0, 16.2]	11.9 [6.0, 22.8]	*p* = 0.68
	No	128	3%				
Atopic (any +)	Yes	138	4%	*p* = 1.0			
	No	32	3%				
# + Aeroallergens (range 0–9)	<6	147	3%	*p* = 0.59			
	≥6	23	4%				
Highest Aeroallergen sIgE level in atopic patients					9.3 [7.9, 43.8]	31.8 [13.7, 71.9]	*p* = 0.20

+ Aeroallergen sIgE is positive if ≥ 3.0 kU/L. Test of between group differences in distributions based on ^a^ Fisher’s exact test or ^b^ Mann–Whitney U text.
